# Tryptophan metabolites and incident cardiovascular disease: The EPIC-Norfolk prospective population study

**DOI:** 10.1016/j.atherosclerosis.2023.117344

**Published:** 2023-10-20

**Authors:** Charlotte J. Teunis, Erik S.G. Stroes, S. Matthijs Boekholdt, Nicholas J. Wareham, Andrew J. Murphy, Max Nieuwdorp, Stanley L. Hazen, Nordin M.J. Hanssen

**Affiliations:** a Department of Internal and Vascular Medicine, Amsterdam University Medical Center, 1105 AZ, Amsterdam, the Netherlands; b Department of Cardiology, Amsterdam University Medical Center, 1105 AZ, Amsterdam, the Netherlands; c MRC Epidemiology Unit, Institute of Metabolic Science, University of Cambridge School of Clinical Medicine, Cambridge, CB2 0QQ, United Kingdom; d Haematopoiesis and Leukocyte Biology, Baker IDI Heart and Diabetes Institute, Melbourne, 3004, Australia; e Department of Immunology, Monash University, Melbourne, 3004, Australia; f Department of Cardiovascular & Metabolic Sciences, and Department of Cardiovascular Medicine, Cleveland Clinic, Cleveland, OH, 44195, USA

**Keywords:** Tryptophan, Metabolites, Cardiovascular Disease, Inflammation, Epidemiology

## Abstract

**Background and aims:**

Cardiovascular disease (CVD) remains the largest cause of death globally due to various risk factors. One novel potential contributor to CVD might be the metabolism of the essential amino acid tryptophan (Trp), which through many pathways can produce immunomodulatory metabolites such as kynurenine, indole-3-propionate and serotonin. We aim to identify the metabolites with the strongest association with cardiovascular disease, utilizing a substantial and diverse cohort of individuals. In our pursuit of this aim, our primary focus is to validate and reinforce the findings from previous cross-sectional studies.

**Methods:**

We used the community-based EPIC-Norfolk cohort (46.3 % men, age 59.8 ± 9.0) with a median follow-up of 22.1 (17.6–23.3) years to study associations between the relative levels of Trp metabolites measured with untargeted metabolomics and incident development of CVD. Serum from n = 11,972 apparently healthy subjects was analysed, of which 6982 individuals had developed CVD at the end of follow-up. Cox proportional hazard models were used to study associations, adjusted for sex, age, conventional cardiovascular risk factors and CRP. All metabolites were Ln-normalised prior to analysis.

**Results:**

Higher levels of Trp were inversely associated with mortality (HR 0.73; CI 0.64–0.83) and fatal CVD (HR 0.76; CI 0.59–0.99). Higher levels of kynurenine (HR 1.33; CI 1.19–1.49) and the [Kynurenine]/[Tryptophan]-ratio (HR 1.24; CI 1.14–1.35) were associated with a higher incident development of CVD. Serotonin was not associated with overall CVD, but we did find associations for myocardial infarction and stroke. Adjustment for CRP did not yield any discernible differences in effect size.

**Conclusions:**

Tryptophan levels were inversely correlated with CVD, while several of its major metabolites (especially kynurenine and serotonin) were positively correlated. These findings indicate that mechanistic studies are required to understand the role of Trp metabolism in CVD with the goal to identify new therapeutic targets.

## 1. Introduction

Cardiovascular disease (CVD) is the leading cause of morbidity and mortality worldwide. The prevalence of individuals living with CVD has nearly doubled from 271 million in 1990 to 523 million in 2019. This is largely explained by the fact that the incidence of conventional CVD risk factors such as type 2 diabetes (T2D), dyslipidaemia, obesity and hypertension has precipitously risen over the past two decades [1,2]. Current CVD prevention strategies are based on lifestyle interventions in combination with medical treatment of conventional risk factors such as dyslipidaemia and hypertension [3]. However, even if recommended risk factor goals can be achieved, the residual risk of CVD remains considerable [4]. This is mainly caused by incomplete understanding of the pathogenesis of CVD events. Therefore, there is an unmet need for the identification of novel etiological factors both in the initiation and progression of CVD, as well as the development of its adverse events.

**Fig. 1.**
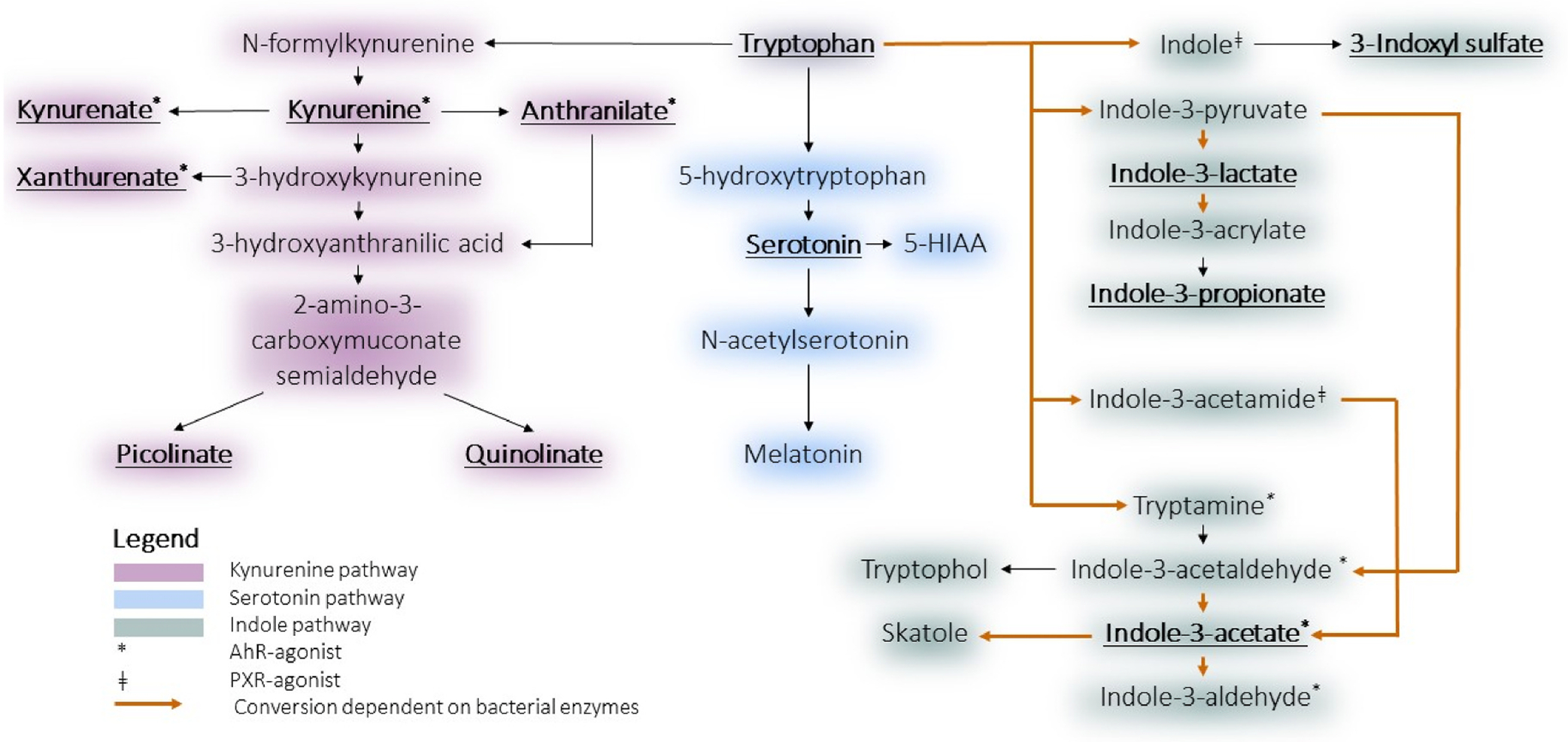
Downstream metabolites of tryptophan metabolism. Pathways involved in tryptophan metabolism. The metabolites that were measured in these pathways, using untargeted metabolomics, are underlined and presented in bold text.

Inflammation plays a critical role in the development of CVD and recent evidence suggests that anti-inflammatory medication reduces the risk of incident CVD [3,5]. Potential novel factors mediating inflammation in CVD include downstream metabolites of the essential amino acid tryptophan (Trp). In mammals, Trp metabolism is predominantly divided into three major pathways: the kynurenine pathway (95%), the indole pathway (5%) and the serotonin/melatonin pathway (1–2%) (Fig. 1) [6]. Multiple downstream metabolites of the kynurenine pathway, such as kynurenine, have been associated with prevalent atherosclerosis [7,8], as well as cardiometabolic diseases such as T2D [9] and obesity [10,11]. The rate-limiting step in the formation of these metabolites is the activity of the enzyme indoleamine 2,3-dioxygenase (IDO). IDO expression is strongly upregulated by proinflammatory molecules such as IFN-γ and interleukins, further increasing concentrations of downstream metabolites [12]. IDO activity can be indirectly estimated as an increased [kynurenine]/[tryptophan]-ratio ([Kyn]/[Trp]-ratio), which is explained by a higher conversion rate of tryptophan into kynurenine [13]. Previous studies have indicated that this increased conversion results in decreased levels of Trp [14,15]. This ratio has been associated with prevalent CVD and conventional risk factors in multiple studies [7,10,16]. Increased concentrations of serotonin, formed in a different Trp pathway, are also associated with atherosclerotic vascular damage [17].

Contrarily, higher concentrations of some end products of the indole pathway produced by gut microbiota have been reported to be associated with a lower risk of atherosclerosis [16]. These metabolites are also decreased in subjects with conventional risk factors. Among these, higher levels of the gut microbiota-generated metabolite indole-3-propionate have been associated with lower rates of T2D and conversely, lower levels of indole-3-propionate have been reported among subjects with obesity [10,18].

Based on preclinical studies, it is hypothesised that low-grade inflammation in CVD causes a shift towards the kynurenine pathway, caused by the upregulation of IDO-activity, and away from indole production, due to perturbations of the gut microbiome [19]. A crucial step towards exploring the relevance of Trp metabolism in CVD is represented by aetiological studies establishing a prospective association between Trp metabolites and incident CVD. Herein we used the European Prospective Investigation into Cancer (EPIC)-Norfolk study with over 11,000 participants and a follow-up time of over 20 years to explore the associations between Trp and its major metabolites with incident development of CVD.

## 2. Patients and methods

The data from EPIC-Norfolk (European Prospective Investigation Into Cancer in the Norfolk Prospective Population Study) will not be made public. Information on how to request data from EPIC-Norfolk can be found online (https://www.epic-norfolk.org.uk/for-researchers/data-sharing/).

### 2.1. Study design and population

EPIC-Norfolk is a prospective population study that included men and women between the age of 39–79 years old in Norfolk, United Kingdom. The baseline survey was from 1993 until 1997. During the first study visit. all participants completed a questionnaire on medical history and lifestyle factors. General measurements were taken and non-fasting blood and urine samples were collected by trained nurses. Characteristics of the cohort are similar to the UK population, although there is a lower proportion of smokers within the EPIC-Norfolk cohort. Details of the study design and population have been published elsewhere [20]. This study exclusively included participants for whom untargeted metabolomics data were available.

### 2.2. Follow-up

During follow-up, patients were flagged for mortality at the UK Office of National Statistics. Vital status was ascertained for the entire cohort and cause of death on death certificates was coded in accordance to the International Classification of Diseases, 10th revision (ICD-10). Furthermore, patients were identified using the National Health Service number linked with the East Norfolk Health Authority (ENCORE), which registers all hospital contacts. All CVD-events were defined by ICD-10 codes I10–I79, ischemic heart disease (IHD) by I20–I25, myocardial infarction (MI) by I21–I22, cerebral infarction by I63, I65 and I66. All stroke events were identified by ICD-10 codes I60–I69 and peripheral artery disease (PAD) by code I70–I79. The study was approved by the Norwich District Health Authority Ethics Committee and, prior to inclusion, all patients gave written informed consent.

### 2.3. Measurement of tryptophan metabolites

After collection, plasma samples were stored in liquid nitrogen at −80 °C. Untargeted metabolomics measurements were performed on non-fasted plasma samples using the Discovery HD4 platform (Metabolon, Morrisville, USA). A detailed description of metabolite measurement has been published elsewhere [21].

### 2.4. Statistical analysis

Descriptive data are presented as number and percentages for categorical variables, as mean with standard deviation for continuous variables with a normal distribution, or as median with interquartile range for continuous variables with a non-normal distribution. The [Kyn]/[Trp]-ratio was calculated as well as the estimated glomerular filtration rate (eGFR) using the chronic kidney disease epidemiology collaboration (CKD-EPI) equation published in 2009 based on creatinine [22]. Variables with a skewed distribution were ln-transformed prior to further analysis (age, triglycerides, CRP, monocytes, granulocytes and all Trp metabolites) to normalise statistical distribution and to reduce the influence of outliers. To assess the differences between groups with and without CVD, a chi-square test was used for categorical variables and a 2-sided *t*-test for continuous variables. Cox proportional hazard regression models were performed to calculate hazard ratios with corresponding 95% confidence intervals (95% CI) for the association between Trp metabolites and incident CVD. Multivariable analysis was adjusted for age, sex (Model 1) and additionally for cardiovascular risk factors (systolic blood pressure, BMI, LDL-cholesterol, triglycerides, smoking status and the presence of diabetes) and eGFR (Model 2), and subsequently we further adjusted for CRP as a marker for low-grade inflammation (Model 3). Missing data were excluded listwise from the analysis. A *p*-value of <0.05 was considered statistically significant. All statistical analysis were performed using SPSS (version 27, Chicago, IL, USA).

## 3. Results

Table 1 summarizes the baseline characteristics of the EPIC-Norfolk participants included in the current analyses. In total, metabolomics measurements were performed in 11,972 individuals. A cardiovascular event occurred in 6989 individuals. Not all metabolites were available in each individual, therefore resulting in a variable number of CVD events per metabolite. Trp was measured in 11,971 participants and anthranilate was the least available metabolite (measured in 3244 participants). We provide an overview of available participants and subsequent CVD events for each metabolite in. Supplementary Tables 1 and 2

### 3.1. The kynurenine pathway

Higher Trp levels were associated with a lower incidence of stroke and IHD over the ensuing 19.5 ± 5.9 years of follow-up after correction for cardiovascular risk factors (Table 2, model 2). These associations remained unaltered after further adjustment for CRP in IHD (Table 2, model 3). We observed a similar trend for incident total CVD, non-fatal MI, non-fatal cerebral infarction and peripheral artery disease (PAD) (Table 2). Higher Trp levels were also associated with lower incident risk of mortality in all three models and a tendency toward lower risk was similarly observed for all fatal cardiovascular events, although this failed to reach significance following adjustments (Table 3).

Elevated kynurenine levels, as well as a higher [Kyn]/[Trp]-ratio, were associated with a greater risk of developing (overall) CVD. These associations were attenuated after correction for cardiovascular risk factors and CRP (Table 2). We found consistent associations between higher kynurenine levels and a higher [Kyn]/[Trp]-ratio and a higher incidence of PAD (Table 2, Model 1–3). Furthermore, higher kynurenine levels and a higher [Kyn]/[Trp]-ratio were associated with a higher incidence of mortality (Table 3, model 1–3). Higher kynurenine levels were associated with a higher incidence of fatal CVD and fatal IHD across all three models. The same trend was observed for fatal MI, fatal stroke and fatal PAD. Furthermore, there was association between the [Kyn]/[Trp]-ratio and fatal MI as well as for fatal PAD, but this lost statistical significance after further adjustment for cardiovascular risk factors and CRP (Table 3, model 2 and 3). In contrast, after adjustment for cardiovascular risk factors and CRP we observed a significant association between a higher [Kyn]/[Trp]-ratio and fatal stroke (Table 2, model 2 and 3). Lastly, we observed an association between higher levels of the downstream metabolite picolinate and fatal CVD, fatal IHD and fatal PAD (Table 3, models 1–3). The same trend was observed for fatal MI, fatal stroke and fatal cerebral infarction (see Table 3). Overall, we found no evidence that associations differed between men and women (Supplementary Table 3).

### 3.2. The serotonin pathway

We observed a association between higher serotonin levels and incidence of MI, which was significant in all three models (see Table 4). We observed a similar association for stroke. The association between higher serotonin levels and a higher incidence of PAD was significant in model 1, with minor attenuation in models 2 and 3.

When exploring the relationship to fatal events, we noted significant associations across all models between serotonin and mortality, fatal CVD and fatal stroke (Table 5). A trend in the same direction was observed in fatal cerebral infarction and fatal PAD. We found no evidence that associations differed between men and women (Supplementary Table 3).

### 3.3. The indole pathway

Overall, we found no consistent associations between metabolites in the indole pathway and cardiovascular outcomes (Table 5). Higher levels of indole-3-propionate were associated with a lower incidence of IHD and PAD (Table 4, model 1), but lost significance after further adjustment for traditional cardiovascular risk factors and CRP, although the direction of the effect was similar (Table 4, model 2 and 3). This trend was also observed for CVD, MI, stroke and cerebral infarction (Table 4). Indole-3-acetate was associated with PAD in all models, but not in any of the other cardiovascular events.

In fatal events, we observed that higher levels indole-3-propionate were associated with lower all-cause mortality (Table 5, model 1–3). The same direction was also found for fatal CVD and fatal IHD in model 1, but this was not statistically significant in other models (models 1–3). The same trends were observed for fatal MI and fatal PAD, but were not seen in fatal stroke and fatal cerebral infarction (Table 5). Higher levels of indole-3-acetate and indole-3-lactate were associated with fatal cerebral infarction (Table 5). Furthermore, indole-3-lactate levels were associated with mortality, fatal CVD, and fatal IHD (, model 1) but not after correction (Table 5, models 2 and 3). The same trend was seen for fatal MI, fatal stroke and fatal PAD (Table 5). Overall, we found no evidence that associations differed between men and women (Supplementary Table 3) and saw no change in point estimate after excluding participants with prior MI or stroke (data not shown).

## 4. Discussion

A principal finding from this study is the association between increased concentrations of plasma kynurenine, the [Kyn]/[Trp]-ratio and a higher incidence of CVD. We also found that higher levels of Trp were associated with a lower incident rate of CVD. These findings imply that there is a potential role of downstream Trp metabolites from the kynurenine pathway as biomarkers or as causal risk factors for CVD.

### 4.1. Interpretations and clinical implications

#### 4.1.1. The kynurenine pathway

Our findings are consistent with previous studies that demonstrated that kynurenine and the [Kyn]/[Trp]-ratio were associated with a higher incidence of CVD [16,23,24]. However, this is the first time that these associations are found in a large, prospective cohort. Several kynurenine metabolites have been thought to have atherogenic effects by modulating the immune system. Multiple studies have reported that kynurenine modulates the immune system in an aryl hydrocarbon receptor (AhR) dependent manner [25–27] and other metabolites, such as kynurenic acid and xanthurenic acid, are also ligands for AhR, which is expressed throughout the body but mostly in immune and vascular tissue.

We postulate that the association between the kynurenine pathway and incident CVD is further reinforced due to an upregulation of IDO-activity via pro-inflammatory molecules, such as IFN-γ and IL-6, associated with low-grade inflammation in cardiometabolic disease [12]. Low-grade inflammation plays a central role in atherogenesis [28] and inflammatory cytokines such as IL-6 and TNF are associated with increased risk of coronary heart disease [29]. Furthermore, low-grade inflammation is also found in metabolic diseases such as obesity and diabetes, both associated with increased levels of IL-6, IL-10, IFN-γ and TNF. Moreover, various trials have shown that targeting low-grade inflammation reduces the risk of acute cardiovascular events in patients with chronic CVD [3,5]. IDO expression is strongly upregulated by IFN-γ [30] and to a lesser extent by TNF, LPS and interleukins [12]. An upregulation of IDO-activity by inflammatory molecules would result in higher levels of kynurenine and possibly lower levels of Trp, consistent with our findings. Importantly, we did not observe an attenuation of the associations after adjustment for CRP. We are therefore careful not to overinterpret these findings. The fact that our point estimates hardly changes, suggests that the regulation of IDO-activity is not highly dependent on IL-1/IL-6 signalling.

The activation of the kynurenine pathway might result in a depletion or decrease of Trp concentrations [15]. Our findings align with this phenomenon, as we observed decreased levels of Trp in association with a higher risk of CVD, which is consistent with similar trends observed in previous clinical studies [14,31].

#### 4.1.2. The serotonin pathway

Serotonin was associated with fatal CVD in this cohort, which has previously been demonstrated in several studies [32–34]. More specifically, serotonin was associated with MI and stroke within EPIC-Norfolk. The mechanism by which serotonin modulates CVD remains unclear. However, serotonin can increase the coagulant properties of platelets [35] which can directly contribute to CVD through several pathways. Serotonin has been extensively studied in the context of depression, due to the increased risk of CVD in this subpopulation. One of these studies found that in patients with CVD and depression there was a higher serotonin receptor density and an increased platelet response in patients with minor depression, possibly explaining the association between higher levels of serotonin and CVD [36]. To our knowledge, there is no interaction between serotonin and pregnane X receptor (PXR), but serotonin has been described to decrease the ability of ligands to bind to the AhR receptor [37]. The effect of these properties in CVD remains to be elucidated.

#### 4.1.3. The indole pathway

The concept has been raised that there is an optimum balance between kynurenine metabolites (increasing the risk of CVD) and indole metabolites (decreasing the risk of CVD). Indeed studies have demonstrated that higher levels of indole-3-propionate are associated with a decrease in atherosclerosis [16] and that lower levels of indole-3-propionate are associated with CVD risk factors such as obesity [38,39] and diabetes [18,40]. This had led to the theory that increased IDO-activity results in a shift away from the production of metabolites from the indole pathway and results in higher concentrations of kynurenine pathway metabolites [19].

Multiple studies found that metabolites from the indole pathway, specifically indole-3-propionate, modulate the PXR, which asserts an anti-inflammatory effect [41,42] and can induce vasodilation [43]. This could possibly account for the association between lower indole-3-propionate levels and PAD in this cohort.

However, within the EPIC-Norfolk cohort we did not find evident association between lower levels of indole-3-propionate and a higher incidence of CVD.

### 4.2. Strengths and limitations

The EPIC-Norfolk cohort provides a unique opportunity to assess the associations between a range of tryptophan metabolites and CVD in a large community-based population. A significant strength of the cohort is its long-term follow-up for incident development of CVD and other adverse outcomes. However, the EPIC-Norfolk cohort is comprised of participants from general practice clinics and is therefore an relatively healthy cohort compared to cohorts recruited through specialist clinics. As with most large epidemiological studies it is also possible that there is a healthy volunteer bias and although EPIC-Norfolk is similar to the UK population [20], the results might not be applicable to different (sub) populations. Additionally, his study revealed no discernible differences between men and women.

Although another strength of this study is the precise use of ICD-10 codes to identify clinical outcomes, it does influence the observed associations. PAD has a clearly defined clinical presentation and annotation whereas stroke comprises a broader range of clinical symptoms and annotations and is therefore harder to categorize. Thus, it is not possible to distinguish from these data whether we found stronger associations for PAD due to a more precise annotation or if there exists an underlying pathophysiological pathway wherein tryptophan plays a more substantial role specifically in PAD. To calculate the associations between Trp metabolites and CVD we used the available data of untargeted metabolomics. Therefore, due to a varying definition of events, the number in events and number of individuals in which data was available per metabolite, we could not directly compare the strength of the associations with CVD.

Overall, we observed no effect of CRP, since there was no difference between model 2 (age, sex and conventional cardiovascular risk factors) and model 3 (model 2 and CRP) in most associations. For some events, such as fatal cerebral stroke, there was a significantly smaller number of events (Supplementary Tables 1 and 2). This may have led to a decrease in statistical power.

Residual confounding, especially by sociodemographic and lifestyle factors (e.g diet), is an important limitation that should be mentioned. While efforts were made to adjust for conventional cardiovascular risk factors, the influence of unmeasured or imprecisely measured factors remains a concern. These factors might introduce biases that could influence the observed associations.

Furthermore, we performed a large number of statistical tests, which may lead to false positive associations. As summarised by Rothman, we opted not to perform adjustment for multiple testing because of the consistency of results across different end points and models. Furthermore, adjustment for multiple testing comes at the cost of increasing type 2 errors over decreasing type 1 errors [44].

Finally, the analyses performed were untargeted and report only relative levels of predicted candidate metabolites. Replication of these results using quantitative assays designed to monitor specific structural isomers to validate the findings is therefore needed. Moreover, the studies were performed on stored, non-fasted samples that did not necessarily remove all platelets from the plasma. As such, the results of the serotonin analyses as presented may be impacted by platelet granule lysis during sample processing. Although multiple studies reveal an association between serotonin and CVD, there are technical considerations to take into account when interpreting these results. Circulation serotonin is mostly found in platelets. Therefore, collection and storage of the samples affect the release of serotonin from platelets [45].

Finally, while processing delay (duration of storage at −80 °C) does not seem to affect metabolite concentrations, the reproducibility of analyte analyses were not tested beyond 2.5 years [46,47] and within EPIC-Norfolk baseline samples were used for metabolomics analysis [21]. Therefore, some samples might have been stored for over 20 years.

### 4.3. Conclusion

**Fig. 2.**
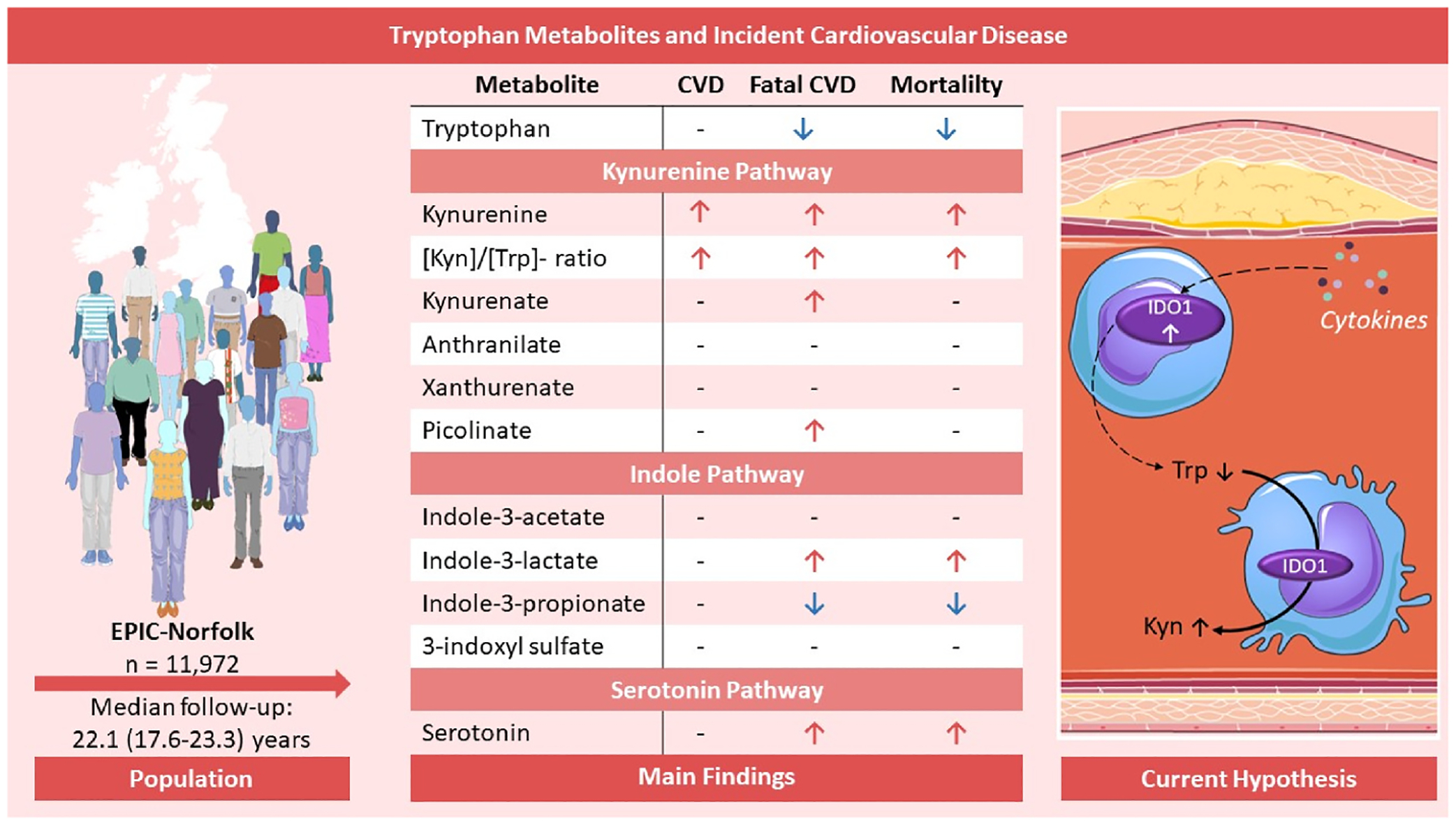
Graphical abstract.

In conclusion, using untargeted mass spectrometry analyses, we report multiple associations between downstream metabolites of tryptophan and incident development of CVD. These findings highlight the potential relevance of tryptophan metabolism in CVD, and argue for further studies to both validate the findings, and identify both underpinning mechanisms and potential therapeutic interventions that leverage targeting of Trp related pathways (Fig. 2).

## Supplementary Material

suppl

Appendix A. Supplementary data

Supplementary data to this article can be found online at https://doi.org/10.1016/j.atherosclerosis.2023.117344.

## Figures and Tables

**Table 1:** Baseline characteristics in the total study population and stratified according to incident cardiovascular events.

	Total	N	No cardiovascular event	Cardiovascular event	*p*-value
Number	11972	11972	4983	6989	–
Years of follow up	22.1 (17.6–23.3)	11972	22.3 (18.7–23.4)	21.9 (17.3–23.2)	<0.05
Male (n)	46.3 (5539)	11972	43.2 (2155)	48.4 (3384)	<0.05
Age (years)	59.8 ± 9.0	11972	56.6 ± 8.8	62.0 ± 8.4	<0.05
Body mass index (kg/m^2^)	26.2 ± 3.8	11956	25. ± 3.5	26.7 ± 3.8	<0.05
Prior MI (n)	3.1 (367)	11959	1.5 (77)	4.2 (290)	<0.05
Prior stroke (n)	1.4 (165)	11963	1.0 (51)	1.6 (114)	<0.05
Smoking status	11876	11876	4951	6925	
- Current smoker (n)	11.3 (1343)	1343	11.4 (562)	11.3 (781)	<0.05
- Former smoker (n)	43 (5105)	5105	39.5 (1957)	45.5 (3148)	<0.05
- Never (n)	45.7 (5428)	5428	49.1 (2432)	43.3 (2996)	<0.05
Diabetes mellitus (n)	2.5 (298)	11959	1.2 (60)	3.4 (239)	<0.05
Systolic blood pressure (mmHg)	136 ± 18	11951	130 ± 17	140 ± 18	<0.05
Diastolic blood pressure (mmHg)	82 ± 11	11951	79 ± 10	85 ± 11	<0.05
eGFR (mL/min/1.73m^2^)	72.9 ± 16.0	10184	75.1 ± 15.7	71.4 ± 16.0	<0.05
Glucose (mmol/L)	4.3 ± 1.7	10270	4.2 ± 1.4	4.4 ± 1.8	<0.05
Cholesterol (mmol/l)	6.2 ± 1.2	11832	6.0 ± 1.1	6.3 ± 1.2	<0.05
LDL-cholesterol (mmol/l)	4.0 ± 1.1	11389	3.9 ± 1.0	4.1 ± 1.1	<0.05
HDL-cholesterol (mmol/l)	1.4 ± 0.4	11389	1.5 ± 0.4	1.4 ± 0.4	<0.05
Triglycerides (mmol/l)	1.5 (1.1–2.2)	11830	1.4 (1.0–2.0)	1.7 (1.3–2.4)	<0.05
CRP (mg/L)	1.6 (0.8–3.3)	10170	1.3 (0.6–2.7)	1.8 (0.9–3.7)	<0.05
HbA1C (%)	5.4 ± 0.9	2929	5.3 ± 0.7	5.5 ± 1.0	<0.05
HbA1C (mmol/mol)	35.6 ± 9.4	2929	34.2 ± 7.7	36.7 ± 10.5	<0.05
White blood count (10^^^3/mcl)	6.6 ± 1.9	7482	6.5 ± 1.8	6.7 ± 1.8	<0.05
Monocyte count (% of TBV)	6.9 (4.6–10.2)	7352	6.6 (4.4–9.8)	7.0 (4.7–10.4)	<0.05
Granulocyte count (% of TBV)	3.8 (3.1–4.8)	7385	3.8 (3.0–4.7)	3.9 (3.1–4.8)	<0.05
Lymphocyte count (% of TBV)	2.0 ± 0.8	7479	2.0 ± 0.8	2.0 ± 0.8	<0.05
Platelet count (10^^^9/L)	254.9 ± 64.0	7485	254.0 ± 61.9	255.7 ± 65.5	0.24
Tryptophan^a^	1.01 ± 0.17	11971	1.01 ± 0.17	1.01 ± 0.17	0.89
Kynurenine^a^	1.04 ± 0.30	11966	1.01 ± 0.29	1.07 ± 0.30	<0.05
[Kyn]/[Trp]-ratio^a^	1.05 ± 0.32	11966	1.01 ± 0.30	1.08 ± 0.33	<0.05
Kynurenate^a^	1.08 ± 0.60	11944	1.05 ± 0.58	1.11 ± 0.62	<0.05
Anthranilate^a^	1.00 (0.75–1.36)	3244	0.97 (0.73–1.33)	1.02 (0.77–1.38)	<0.05
Xanthurenate^a^	1.00 (0.70–1.40)	10420	1.00 (0.70–1.40)	1.00 (0.71–1.40)	0.42
Picolinate^a^	1.00 (0.78–1.27)	5835	1.01 (0.79–1.27)	1.00 (0.77–1.27)	0.61
Indole-3-acetate^a^	1.00 (0.77–1.35)	11970	0.99 (0.77–1.32)	1.01 (0.77–1.37)	<0.05
Indole-3-lactate^a^	1.00 (0.83–1.22)	11970	0.97 (0.81–1.18)	1.02 (0.85–1.24)	<0.05
Indole-3-propionate^a^	1.00 (0.65–1.54)	11895	1.01 (0.66–1.55)	1.00 (0.64–1.52)	<0.05
3-indoxyl sulfate^a^	1.00 (0.69–1.38)	11970	0.98 (0.68–1.35)	1.01 (0.70–1.41)	<0.05
Serotonin^a^	1.00 (0.61–1.61)	9381	1.00 (0.62–1.60)	1.00 (0.61–1.61)	0.97

MI; myocardial infarction, TBV; total blood volume. Data are presented as number and percentages for categorical variables, as mean with standard deviation for continuous variables with a normal distribution, or as median with interquartile range for continuous variables with a non-normal distribution. Differences were tested with a Chi-square or a student’s T-test, as appropriate. Skewed variables were Ln-normalised prior to analyses.

a Measured with untargeted metabolomics.

**Table 2:** Associations between the kynurenine pathway metabolites and total incident cardiovascular disease.

Metabolite	M	CVD	IHD	MI	Stroke (all)	Cerebral infarction	PAD
**Tryptophan**	1	0.96 (0.84–1.11)	0.83 (0.67–1.02)	0.99 (0.62–1.58)	**0.76 (0.59–0.99)**	0.84 (0.56–1.26)	0.88 (0.61–1.27)
	2	0.93 (0.81–1.07)	**0.79 (0.65–0.96)**	0.93 (0.59–1.47)	**0.76 (0.58–0.99)**	0.84 (0.55–1.28)	0.88 (0.60–1.27)
	3	0.95 (0.82–1.09)	**0.81 (0.66–0.99)**	0.96 (0.60–1.54)	**0.76 (0.58–1.00)**	0.84 (0.55–1.29)	0.92 (0.62–1.35)
**Kynurenine**	1	**1.33 (1.19–1.49)**	**1.28 (1.06–1.55)**	1.08 (0.76–1.54)	1.16 (0.91–1.48)	0.96 (0.68–1.36)	**1.80 (1.35–2.40)**
	2	**1.14 (1.02–1.28)**	1.12 (0.92–1.37)	1.05 (0.73–1.51)	1.14 (0.89–1.47)	0.94 (0.65–1.36)	**1.69 (1.26–2.28)**
	3	**1.12 (1.00–1.26)**	1.09 (0.90–1.33)	1.02 (0.71–1.47)	1.13 (0.88–1.45)	0.94 (0.65–1.35)	**1.01 (1.21–2.20)**
**[Kyn]/[Trp]-ratio**	1	**1.24 (1.14–1.35)**	**1.32 (1.16–1.51)**	1.08 (0.82–1.42)	**1.20 (1.00–1.43)**	1.06 (0.81–1.39)	**1.61 (1.34–1.95)**
	2	**1.13 (1.04–1.23)**	**1.22 (1.06–1.39)**	1.07 (0.81–1.41)	1.18 (0.98–1.41)	1.04 (0.79–1.38)	**1.51 (1.24–1.83)**
	3	**1.11 (1.02–1.21)**	**1.17 (1.02–1.34)**	1.03 (0.78–1.36)	1.16 (0.96–1.39)	1.04 (0.78–1.38)	**1.44 (1.18–1.75)**
**Kynurenate**	1	**1.18 (1.09–1.27)**	**1.13 (1.00–1.28)**	0.99 (0.79–1.25)	0.97 (0.82–1.14)	0.91 (0.72–1.16)	1.14 (0.94–1.39)
	2	1.04 (0.96–1.12)	1.02 (0.90–1.16)	0.95 (0.75–1.22)	0.92 (0.77–1.09)	0.87 (0.68–1.11)	1.05 (0.86–1.29)
	3	1.04 (0.97–1.13)	1.02 (0.90–1.17)	0.96 (0.75–1.23)	0.92 (0.78–1.10)	0.87 (0.68–1.11)	1.07 (0.87–1.30)
**Anthranilate**	1	1.09 (0.97–1.22)	1.15 (0.95–1.39)	1.09 (0.77–1.54)	1.09 (0.85–1.39)	1.32 (0.93–1.86)	1.33 (0.99–1.78)
	2	1.07 (0.96–1.20)	1.13 (0.94–1.37)	1.11 (0.78–1.58)	1.11 (0.86–1.42)	1.35 (0.95–1.91)	**1.34 (1.00–1.80)**
	3	1.07 (0.96–1.20)	1.13 (0.93–1.37)	1.11 (0.78–1.58)	1.11 (0.86–1.42)	1.35 (0.95–1.91)	**1.34 (1.00–1.81)**
**Xanthurenate**	1	**1.10 (1.04–1.16)**	**1.14 (1.04–1.25)**	1.06 (0.90–1.26)	1.01 (0.89–1.13)	0.98 (0.83–1.17)	1.02 (0.89–1.17)
	2	**1.06 (1.01–1.12)**	**1.10 (1.00–1.21)**	1.05 (0.88–1.25)	0.99 (0.88–1.12)	0.98 (0.82–1.16)	0.99 (0.87–1.14)
	3	**1.07 (1.01–1.13)**	**1.10 (1.01–1.22)**	1.06 (0.89–1.26)	1.00 (0.89–1.13)	0.98 (0.83–1.16)	1.01 (0.88–1.16)
**Picolinate**	1	0.97 (0.88–1.06)	1.16 (0.99–1.36)	1.05 (0.79–1.40)	0.98 (0.79–1.20)	1.15 (0.86–1.55)	1.22 (0.96–1.54)
	2	1.04 (0.95–1.14)	**1.24 (1.06–1.45)**	1.10 (0.83–1.47)	0.97 (0.79–1.20)	1.17 (0.87–1.57)	**1.28 (1.01–1.61)**
	3	1.05 (0.95–1.15)	**1.25 (1.07–1.46)**	1.11 (0.83–1.49)	0.98 (0.79–1.21)	1.17 (0.87–1.57)	**1.30 (1.03–1.64)**

Data were analysed using cox proportional hazard regression analysis. HRs with 95%CIs are reported per 1 Ln change in metabolite. Model 1: age and sexModel 2: model 1 + systolic blood pressure, BMI, LDL-cholesterol, triglycerides, smoking status, the presence of diabetes and eGFR. Model 3: model 2+ CRP. Numbers in bold indicate statistical significance at *p* < 0.05.

**Table 3:** Associations between the kynurenine pathway metabolites and fatal cardiovascular disease.

Metabolite	M	Mortality	Fatal CVD	Fatal IHD	Fatal MI	Fatal stroke (all)	Fatal cerebral infarction	Fatal PAD
**Tryptophan**	1	**0.73 (0.64–0.83)**	**0.76 (0.59–0.99)**	0.74 (0.51–1.07)	0.79 (0.41–1.50)	0.89 (0.52–1.51)	0.89 (0.19–4.20)	0.90 (0.32–2.50)
	2	**0.72 (0.63–0.82)**	0.76 (0.58–1.01)	0.73 (0.49–1.10)	0.76 (0.41–1.43)	0.92 (0.51–1.65)	1.01 (0.16–6.48)	0.88 (0.31–2.49)
	3	**0.74 (0.64–0.84)**	0.80 (0.60–1.07)	0.77 (0.50–1.20)	0.81 (0.41–1.61)	0.93 (0.52–1.68)	1.02 (0.16–6.61)	0.93 (0.32–2.73)
**Kynurenine**	1	**1.41 (1.23–1.61)**	**1.49 (1.18–1.90)**	**1.66 (1.16–2.36)**	1.74 (0.99–3.07)	1.21 (0.78–1.88)	0.87 (0.23–3.36)	**2.35 (1.09–5.07)**
	2	**1.33 (1.15–1.53)**	**1.40 (1.09–1.80)**	**1.54 (1.06–2.23)**	1.46 (0.80–2.65)	1.33 (0.84–2.11)	0.91 (0.22–3.78)	1.98 (0.89–4.44)
	3	**1.29 (1.12–1.49)**	**1.36 (1.06–1.75)**	**1.49 (1.03–2.17)**	1.42 (0.78–2.58)	1.32 (0.83–2.10)	0.90 (0.22–3.77)	1.93 (0.86–4.31)
**[Kyn]/[Trp]-ratio**	1	**1.44 (1.32–1.58)**	**1.51 (1.29–1.76)**	**1.62 (1.28–2.05)**	**1.61 (1.11–2.33)**	1.32 (0.98–1.77)	0.95 (0.33–2.70)	**1.73 (1.07–2.80)**
	2	**1.40 (1.27–1.54)**	**1.42 (1.20–1.68)**	**1.49 (1.17–1.90)**	1.39 (0.96–2.02)	**1.38 (1.01–1.88)**	0.92 (0.31–2.76)	1.54 (0.91–2.60)
	3	**1.34 (1.22–1.48)**	**1.37 (1.16–1.62)**	**1.43 (1.12–1.83)**	1.33 (0.91–1.95)	**1.38 (1.01–1.88)**	0.91 (0.30–2.75)	1.48 (0.87–2.53)
**Kynurenate**	1	1.03 (0.94–1.13)	**1.18 (1.00–1.38)**	**1.52 (1.20–1.92)**	1.24 (0.85–1.83)	0.82 (0.60–1.10)	1.19 (0.47–2.97)	1.29 (0.76–2.18)
	2	0.97 (0.88–1.07)	1.03 (0.86–1.22)	**1.32 (1.02–1.71)**	1.01 (0.67–1.53)	0.76 (0.55–1.05)	1.07 (0.40–2.89)	1.15 (0.66–2.00)
	3	0.98 (0.89–1.08)	1.04 (0.87–1.23)	**1.33 (1.03–1.71)**	1.01 (0.70–1.53)	0.76 (0.55–1.05)	1.08 (0.40–2.90)	1.16 (0.66–2.02)
**Anthranilate**	1	1.10 (0.96–1.26)	1.10 (0.86–1.40)	1.13 (0.79–1.63)	0.88 (0.51–1.55)	1.11 (0.73–1.71)	1.17 (0.24–5.87)	0.53 (0.23–1.23)
	2	1.13 (0.98–1.30)	1.08 (0.85–1.38)	1.09 (0.76–1.58)	0.82 (0.46–1.46)	1.13 (0.73–1.76)	1.26 (0.22–7.40)	0.57 (0.24–1.35)
	3	1.13 (0.98–1.30)	1.09 (0.85–1.39)	1.09 (0.76–1.58)	0.82 (0.46–1.45)	1.14 (0.74–1.77)	1.24 (0.21–7.32)	0.57 (0.24–1.35)
**Xanthurenate**	1	1.02 (0.95–1.09)	1.00 (0.89–1.13)	1.16 (0.97–1.38)	1.09 (0.81–1.45)	0.94 (0.76–1.17)	1.21 (0.59–2.48)	0.97 (0.65–1.48)
	2	0.99 (0.92–1.06)	0.97 (0.86–1.10)	1.10 (0.91–1.32)	1.02 (0.76–1.37)	0.95 (0.76–1.19)	1.25 (0.60–2.59)	0.88 (0.59–1.31)
	3	1.00 (0.93–1.07)	0.98 (0.87–1.11)	1.11 (0.93–1.34)	1.03 (0.77–1.39)	0.96 (0.77–1.19)	1.26 (0.61–2.61)	0.90 (0.60–1.34)
**Picolinate**	1	1.05 (0.94–1.18)	**1.25 (1.02–1.53)**	**1.36 (1.02–1.82)**	1.02 (0.63–1.67)	1.28 (0.88–1.86)	2.05 (0.57–7.41)	**1.87 (1.06–3.31)**
	2	1.07 (0.95–1.20)	**1.29 (1.06–1.58)**	**1.43 (1.07–1.91)**	1.10 (0.68–1.80)	1.27 (0.87–1.85)	2.15 (0.63–7.39)	**1.87 (1.12–3.13)**
	3	1.08 (0.96–1.21)	**1.30 (1.07–1.59)**	**1.44 (1.08–1.92)**	1.11 (0.68–1.81)	1.27 (0.88–1.86)	2.17 (0.63–7.46)	**1.91 (1.15–3.20)**

Data were analysed using cox proportional hazard regression analysis. HRs with 95%CIs are reported per 1 Ln change in metabolite. Model 1: age and sex. Model 2: model 1 + systolic blood pressure, BMI, LDL-cholesterol, triglycerides, smoking status, the presence of diabetes and eGFR. Model 3: model 2+ CRP. Numbers in bold indicate statistical significance at *p* < 0.05.

**Table 4:** Associations between the indole & serotonin pathway metabolites and incident total cardiovascular disease.

Metabolite	M	CVD	IHD	MI	Stroke (all)	Cerebral infarction	PAD
**Indole-3-acetate**	1	0.99 (0.93–1.05)	1.06 (0.96–1.17)	1.16 (0.98–1.39)	0.92 (0.81–1.05)	1.05 (0.88–1.26)	**1.20 (1.04–1.39)**
	2	1.00 (0.94–1.06)	1.08 (0.98–1.20)	1.18 (0.99–1.41)	0.94 (0.82–1.06)	1.09 (0.91–1.30)	**1.25 (1.08–1.44)**
	3	1.00 (0.95–1.06)	1.09 (0.99–1.20)	1.19 (1.00–1.41)	0.94 (0.83–1.07)	1.09 (0.91–1.30)	**1.25 (1.08–1.44)**
**Indole-3-lactate**	1	**1.17 (1.07–1.29)**	1.05 (0.89–1.23)	0.86 (0.64–1.16)	1.04 (0.84–1.28)	1.01 (0.75–1.37)	**1.36 (1.07–1.74)**
	2	1.02 (0.92–1.12)	0.88 (0.75–1.04)	**0.74 (0.54–1.00)**	0.94 (0.76–1.17)	0.94 (0.69–1.28)	1.14 (0.88–1.46)
	3	1.02 (0.93–1.13)	0.90 (0.76–1.06)	0.75 (0.55–1.01)	0.95 (0.76–1.18)	0.94 (0.69–1.28)	1.15 (0.90–1.48)
**Indole-3-propionate**	1	0.98 (0.95–1.02)	**0.93 (0.87–0.99)**	0.97 (0.86–1.09)	0.94 (0.87–1.03)	0.99 (0.87–1.11)	**0.84 (0.77–0.93)**
	2	1.02 (0.98–1.06)	0.97 (0.91–1.03)	1.01 (0.89–1.14)	0.96 (0.88–1.04)	1.02 (0.90–1.15)	**0.90 (0.82–0.99)**
	3	1.02 (0.98–1.06)	0.98 (0.92–1.04)	1.02 (0.90–1.15)	0.96 (0.89–1.05)	1.02 (0.90–1.15)	**0.91 (0.82–1.00)**
**3-indoxyl sulfate**	1	1.01 (0.96–1.06)	1.03 (0.95–1.12)	0.89 (0.77–1.03)	0.93 (0.84–1.04)	1.00 (0.86–1.17)	1.10 (0.97–1.25)
	2	1.02 (0.97–1.07)	1.02 (0.94–1.11)	0.88 (0.76–1.02)	0.95 (0.85–1.06)	1.05 (0.89–1.23)	1.13 (0.99–1.29)
	3	1.02 (0.97–1.07)	1.02 (0.94–1.12)	0.88 (0.76–1.02)	0.95 (0.85–1.06)	1.05 (0.89–1.23)	1.13 (0.99–1.29)
**Serotonin**	1	1.02 (0.98–1.06)	1.05 (0.98–1.13)	**1.17 (1.02–1.33)**	**1.11 (1.01–1.21)**	1.12 (0.98–1.27)	**1.12 (1.01–1.25)**
	2	1.03 (0.99–1.07)	1.06 (0.99–1.14)	**1.16 (1.02–1.32)**	**1.10 (1.01–1.21)**	1.11 (0.98–1.26)	1.10 (0.99–1.23)
	3	1.03 (0.99–1.07)	1.06 (0.99–1.14)	**1.16 (1.02–1.32)**	**1.10 (1.01–1.21)**	1.11 (0.98–1.26)	1.10 (0.99–1.22)

Data were analysed using cox proportional hazard regression analysis. HRs with 95%CIs are reported per 1 Ln change in metabolite. Model 1: age and sex. Model 2: model 1 + systolic blood pressure, BMI, LDL-cholesterol, triglycerides, smoking status, the presence of diabetes and eGFR. Model 3: model 2+ CRP. Numbers in bold indicate statistical significance at *p* < 0.05.

**Table 5:** Associations between the indole & serotonin pathway metabolites and fatal cardiovascular disease.

Metabolite	M	Mortality	Fatal CVD	Fatal IHD	Fatal MI	Fatal stroke (all)	Fatal cerebral infarction	Fatal PAD
**Indole-3-acetate**	1	1.01 (0.94–1.09)	1.06 (0.94–1.20)	1.07 (0.89–1.28)	0.95 (0.71–1.29)	0.95 (0.75–1.20)	0.95 (0.75–1.20)	1.38 (0.94–2.02)
	2	1.03 (0.96–1.10)	1.08 (0.95–1.22)	1.07 (0.90–1.29)	0.96 (0.71–1.29)	0.97 (0.77–1.23)	0.97 (0.77–1.23)	1.42 (0.98–2.06)
	3	1.04 (0.97–1.11)	1.08 (0.96–1.22)	1.08 (0.90–1.29)	0.96 (0.71–1.30)	0.98 (0.77–1.23)	0.98 (0.77–1.23)	1.43 (0.99–2.07)
**Indole-3-lactate**	1	**1.17 (1.04–1.32)**	**1.32 (1.08–1.63)**	**1.50 (1.11–2.02)**	1.49 (0.92–2.43)	1.28 (0.87–1.87)	**5.83 (2.06–16.54)**	1.57 (0.81–3.06)
	2	1.07 (0.95–1.21)	1.15 (0.93–1.43)	1.23 (0.90–1.68)	1.18 (0.71–1.96)	1.28 (0.86–1.89)	**5.86 (1.96–17.50)**	1.15 (0.58–2.28)
	3	1.10 (0.97–1.24)	1.18 (0.96–1.47)	1.27 (0.93–1.73)	1.22 (0.74–2.03)	1.28 (0.86–1.90)	**5.87 (1.97–17.53)**	1.18 (0.59–2.33)
**Indole-3-propionate**	1	**0.86 (0.82–0.90)**	**0.90 (0.83–0.98)**	**0.86 (0.76–0.97)**	0.86 (0.71–1.05)	1.02 (0.87–1.19)	1.18 (0.73–1.92)	0.79 (0.61–1.03)
	2	**0.89 (0.85–0.93)**	0.93 (0.85–1.01)	0.89 (0.78–1.00)	0.89 (0.73–1.09)	1.03 (0.88–1.20)	1.16 (0.72–1.88)	0.86 (0.66–1.12)
	3	**0.90 (0.85–0.94)**	0.94 (0.86–1.02)	0.90 (0.79–1.02)	0.91 (0.74–1.11)	1.03 (0.88–1.21)	1.17 (0.72–1.88)	0.87 (0.67–1.13)
**3-indoxyl sulfate**	1	1.00 (0.94–1.06)	1.08 (0.97–1.21)	1.09 (0.93–1.28)	1.00 (0.77–1.29)	1.11 (0.91–1.37)	1.17 (0.72–1.89)	0.97 (0.68–1.37)
	2	1.01 (0.95–1.08)	1.09 (0.98–1.22)	1.07 (0.91–1.27)	0.95 (0.73–1.24)	1.19 (0.96–1.47)	0.68 (0.40–1.16)	0.99 (0.70–1.41)
	3	1.02 (0.95–1.08)	1.10 (0.98–1.23)	1.08 (0.91–1.27)	0.95 (0.73–1.24)	1.19 (0.96–1.47)	0.69 (0.39–1.20)	0.99 (0.70–1.41)
**Serotonin**	1	**1.07 (1.02–1.13)**	**1.10 (1.00–1.20)**	1.05 (0.91–1.20)	0.98 (0.78–1.23)	**1.22 (1.03–1.44)**	1.39 (0.87–2.23)	1.21 (0.90–1.61)
	2	**1.07 (1.01–1.12)**	**1.10 (1.00–1.21)**	1.05 (0.92–1.21)	1.01 (0.81–1.26)	**1.20 (1.02–1.42)**	1.39 (0.87–2.23)	1.20 (0.90–1.61)
	3	**1.07 (1.01–1.12)**	**1.10 (1.00–1.21)**	1.06 (0.92–1.21)	1.01 (0.81–1.27)	**1.20 (1.02–1.42)**	1.39 (0.87–2.23)	1.20 (0.90–1.61)

Data were analysed using cox proportional hazard regression analysis. HRs with 95%CIs are reported per 1 Ln change in metabolite. Model 1: age and sex. Model 2: model 1 + systolic blood pressure, BMI, LDL-cholesterol, triglycerides, smoking status, the presence of diabetes and eGFR. Model 3: model 2+ CRP. Numbers in bold indicate statistical significance at *p* < 0.05.
