# Supplementary material for: Tryptophan metabolites and incident cardiovascular disease: The EPIC-Norfolk prospective population study
Source: Atherosclerosis. Author manuscript; Available in PMC 2025 Oct 10. (PMC12512466; doi:10.1016/j.atherosclerosis.2023.117344)
Supplement: suppl [file NIHMS2112542-supplement-suppl.pdf]

Supplementary Data

Supplementary table 1. Numbers of non-fatal events and individuals analysed per metabolite

Metabolite	***Type of Event***					
	**CVD**	**IHD**	**MI**	**Stroke**	**Cerebral Infarction**	**PAD**
**Tryptophan**	5448/9487	1885/9469	553/9463	1128/9482	543/9482	814/9471
**Kynurenine**	5445/9483	1885/9465	553/9459	1125/9478	542/9478	813/9467
**[Kyn]/[Trp]-ratio**	5445/9483	1885/9465	553/9459	1125/9478	542/9478	813/9467
**Kynurenate**	5438/9471	1882/9453	551/9447	1123/9466	541/9466	811/9455
**Anthranilate**	1528/1085	546/2598	159/2592	317/2574	162/2564	223/2598
**Xanthurenate**	4755/8267	1641/8252	486/8247	971/8264	471/8264	726/8254
**Picolinate**	2699/4701	941/4683	279/4674	542/4660	268/4643	410/4683
**Indole-3-acetate**	5447/9486	1885/9468	553/9462	1127/9481	543/9481	814/9470
**Indole-3-lactate**	5447/9486	1885/9468	553/9462	1127/9481	543/9481	814/9470
**Indole-3-propionate**	5413/9431	1875/9413	549/9407	1123/9426	541/9426	810/9415
**3-indoxyl-sulfate**	5447/9486	1885/9468	553/9462	1127/9481	543/9481	814/9470
**Serotonin**	4244/7458	1464/7447	433/7443	874/7456	434/7456	640/7448

Supplementary table 2. Number of fatal events and individuals analysed per metabolite

Metabolite	***Type of Event***						
	**Mortality**	**CVD**	**IHD**	**MI**	**Stroke**	**Cerebral Infarction**	**PAD**
**Tryptophan**	3495/9487	1114/9487	512/9487	194/9487	329/9483	35/9390	105/9478
**Kynurenine**	3493/9483	1113/9483	512/9483	194/9483	328/9479	35/9386	105/9474
**[Kyn]/[Trp]-ratio**	3493/9483	1113/9483	512/9483	194/9483	328/9479	35/9386	105/9474
**Kynurenate**	3490/9471	1112/9471	511/9471	194/9471	328/9467	35/9374	105/9462
**Anthranilate**	992/2613	333/2613	145/2613	64/2596	106/2603	8/2580	29/2610
**Xanthurenate**	2989/8267	958/8267	444/8267	165/8267	281/8265	27/8186	86/8261
**Picolinate**	1748/4701	556/4701	251/4701	98/4690	165/4690	11/4650	50/4697
**Indole-3-acetate**	3494/9486	1114/9486	512/9486	194/9486	329/9482	35/9389	105/9477
**Indole-3-lactate**	3494/9486	1114/9486	512/9486	194/9486	329/9482	35/9389	105/9477

**Indole-3-propionate**	3467/9431	1106/9431	508/9431	193/9431	327/9427	35/9336	105/9422
**3-indoxyl-sulfate**	3494/9486	1114/9486	512/9486	194/9486	329/9482	35/9389	105/9477
**Serotonin**	2739/7458	826/7458	381/7458	143/7458	259/7388	31/7384	85/7453

Supplementary table 3. Interaction analysis for sex

**Metabolite**	**Type of event**												
	Mortality	*CVD*	*Fatal CVD*	*IHD*	*Fatal IHD*	*MI*	*Fatal MI*	*Stroke*	*Fatal stroke*	*Cerebral infarct*	*Fatal cerebral infarction*	*PAD*	*Fatal PAD*
**Tryptophan**	0.35	0.25	**0.04**	0.19	**0.05**	0.12	**0.00**	0.43	0.29	0.73	0.72	0.82	0.07
**Kynurenine**	0.89	0.79	0.44	0.24	0.93	**0.03**	0.21	0.95	**0.01**	0.90	0.55	0.35	0.74
**[Kyn]/[Trp]-ratio**	0.22	0.60	0.28	0.86	0.10	0.42	0.28	1.00	0.15	0.49	0.40	0.77	0.12
**Kynurenate**	0.99	0.21	0.59	0.96	0.62	**0.00**	**0.04**	0.43	0.18	0.82	0.40	0.39	0.95
**Anthranilate**	0.94	0.30	0.71	0.71	0.78	0.64	0.20	0.30	0.51	0.65	0.15	0.09	0.80
**Xanthurenate**	0.51	**0.01**	0.47	0.24	0.75	0.68	0.28	0.11	0.28	0.07	0.95	0.38	0.61
**Picolinate**	0.41	0.13	0.38	0.10	0.42	0.83	0.93	0.90	0.85	0.91	0.76	0.14	0.46
**Indole-3-acetate**	0.93	0.55	0.49	0.67	0.96	0.35	0.08	0.77	0.43	0.80	0.58	0.36	0.71
**Indole-3-lactate**	0.22	0.46	0.12	0.30	0.43	0.01	**0.03**	0.41	0.03	0.57	0.08	0.39	0.78
**Indole-3-propionate**	0.12	0.96	0.52	0.05	0.47	0.11	0.26	0.11	0.75	0.30	0.99	0.63	0.16
**3-indoxyl-sulfate**	0.70	0.89	0.96	0.81	0.38	0.53	0.45	0.55	0.94	**0.01**	0.80	0.11	0.80
**Serotonin**	0.13	0.10	0.68	0.56	0.34	0.61	0.07	0.56	0.15	0.87	0.79	0.40	0.30

*Interaction between sex and tryptophan metabolite (interaction term sex*metabolite) was investigated in a Cox proportional hazards model adjusted for sex, age, the metabolite of interest, systolic blood pressure, BMI, LDL-cholesterol, triglycerides, smoking status and the presence of diabetes. Numbers is bold highlight significance at $P_{interaction}$<0.*
